# Supplementary material for: Tumorigenicity decrease in Bcl-xL deficient MDCK cells ensuring the safety for influenza vaccine production
Source: PLoS One. 2024 Dec 16;19(12):e0311069. doi: 10.1371/journal.pone.0311069 (PMC11649150; doi:10.1371/journal.pone.0311069)
Supplement: S1 File — (ZIP) [file pone.0311069.s002.zip › S2_File/4_KOG_enrichment/network/B_vs_A.up_KOG_enrichment_network.pdf]

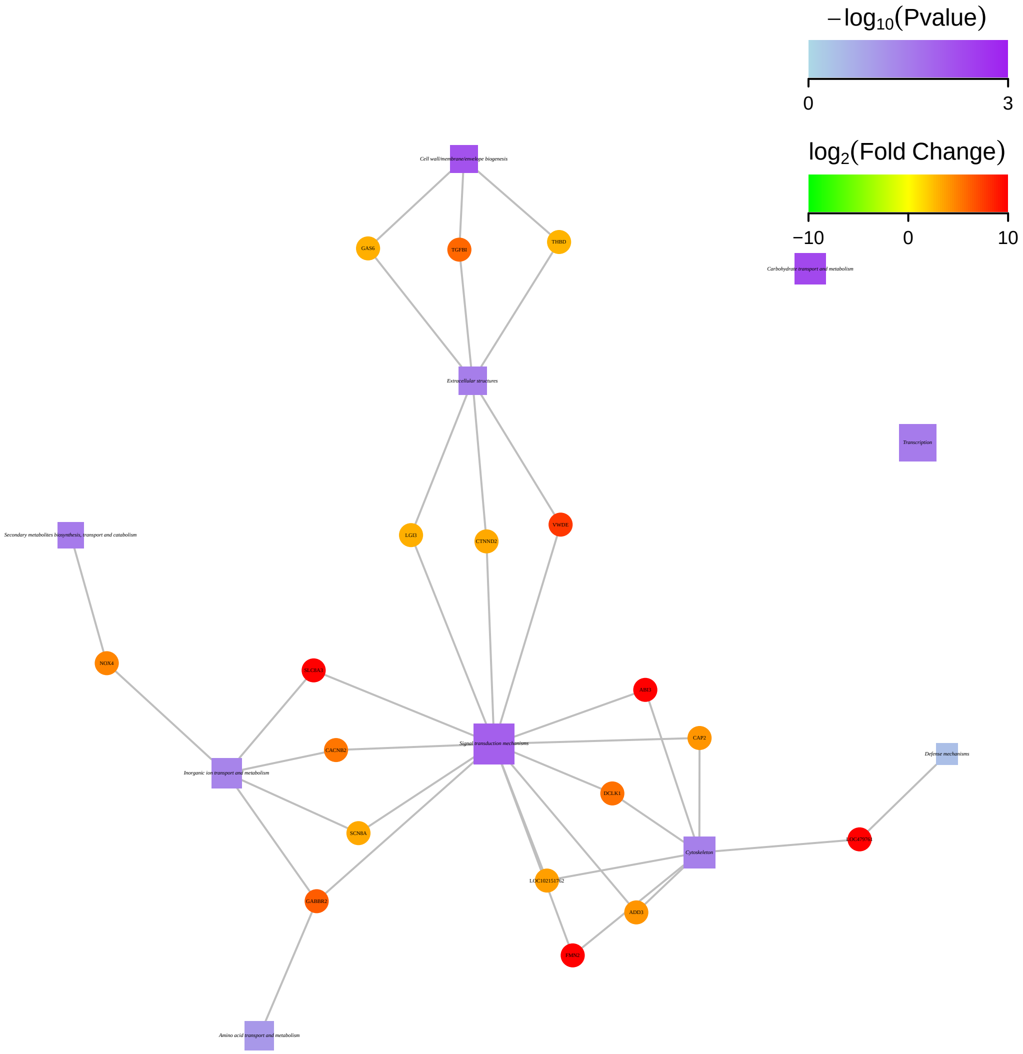

−log10(Pvalue)
0
3
log2(Fold Change)
−10
0
10
Cell wall/membrane/envelope biogenesis
GAS6
TGFBI
THBD
Carbohydrate transport and metabolism
Extracellular structures
Transcription
Secondary metabolites biosynthesis, transport and catabolism
LGI3
CTNND2
VWDE
NOX4
SLC8A3
ABI3
CAP2
Signal transduction mechanisms
CACNB2
Inorganic ion transport and metabolism
DCLK1
Defense mechanisms
SCN8A
Cytoskeleton
LOC479761
LOC102151762
GABBR2
ADD3
FMN2
Amino acid transport and metabolism
